# Effect of Physical Activity on Depression in Patients with Parkinson’s Disease: A Systematic Review and Meta-Analysis

**DOI:** 10.3390/ijerph19116849

**Published:** 2022-06-03

**Authors:** Jianing Tian, Yujie Kang, Peifeng Liu, Hongyan Yu

**Affiliations:** 1 Department of Physical Education, Shanghai Jiao Tong University, Shanghai 200240, China; 1253614982@sjtu.edu.cn (J.T.); yujie_kang@163.com (Y.K.); 2 Department of Physical Education, Central South University, Changsha 410083, China; liupeifeng@csu.edu.cn

**Keywords:** Parkinson’s disease, physical activity, depression, meta-analysis

## Abstract

Parkinson’s disease (PD) is the second most common neurodegenerative disease worldwide, and approximately 50% of PD patients suffer from depression. We aim to determine the effects of physical activity on depression in PD patients and to provide scientific evidence-based exercise prescriptions for PD patients. A systematic review was conducted by searching PubMed, Embase, Cochrane Library, and PsycInfo until February 2022 for randomized controlled trial (RCT) studies published in English. The primary outcome was a score on a depression scale. A total of 14 RCTs involving 516 patients with PD were included in this study. The results of the meta-analysis showed that physical activity had a moderate and significant improvement in depression in PD patients (SMD = −0.60; 95% CI = −0.79 to −0.41; *p* < 0.00001). Subgroup analysis indicated that resistance exercise for 60–90 min more than 4 times per week for up to 12 weeks had a significant effect on PD patients who have had the disease for more than 5 years. Meta-regression showed that intervention type, intervention time, intervention frequency, intervention period, age, and disease duration were not sources of heterogeneity. Physical activity may reduce depression in PD patients. However, other larger sample sizes and high-quality studies are needed to validate these effects in the future.

## 1. Introduction

Parkinson’s disease (PD) is a common chronic neurodegenerative disease [1]. The degeneration of dopaminergic (DA) neurons in the substantia nigra of the midbrain is the most major pathogenic change in Parkinson’s disease, which results in a large drop in striatal DA content and causes the disease [2]. PD affects the physical, psychological, social, and functional states of the person. It is the second most prevalent neurodegenerative disease in the world after Alzheimer’s disease [3]. The prevalence of PD is positively correlated with age, and it affects approximately 1% of the global population over the age of 65 [4]. Currently, given the serious aging of the world’s population [5], the number of people suffering from Parkinson’s disease will also increase significantly. The motor symptoms of PD are manifested by bradykinesia, muscle rigidity, resting tremors, and postural instability. Cognitive impairment, depression and anxiety, and fatigue are the prevalent nonmotor symptoms in PD patients [6]. Treatment of mental health issues is generally overlooked in comparison with motor symptoms, especially depression, which is the most commonly seen nonmotor symptom [7]. It Affects approximately 50% of PD patients [8]. Depression negatively affects interpersonal functioning, quality of life, and well-being in PD patients, which also contributes to a higher mortality rate in PD patients [9]. In clinical diagnosis, it is difficult to distinguish depression from PD because people suffering from depression will show lack of happiness, worthlessness, loss of appetite, sleep disturbance, and psychomotor retardation. These symptoms will also be shown in PD patients who are not diagnosed with depressive mood [10]. The Ravina B (2007) clinical trial found that depression was common in early PD patients, but was generally untreated [11]. Clinical treatment of depression in PD patients is currently most commonly administered through selective serotonin reuptake inhibitor (SSRI) antidepressants [12]. However, medication in general has side effects, which is the popularly received view. Some studies have found that medication with SSRIs often causes patients to experience adverse events, such as headache, nausea, sweating [13], increased tremor [14], increased fatigue, and constipation [15]. Additionally, there is no strong evidence to support the efficacy of SSRIs for depression in patients with PD [16]. Some studies suggest that SSRIs are no more effective than placebos in treating depression in PD patients [12,17]. Given the above, it is imperative to find other effective treatment options.

In recent years, there has been a growing body of research on the effects of physical activity on depression in people with PD, and some review-type studies have explored the effects of different exercise programs, such as yoga [18], tai chi and qigong [19], and dance [20], on the intervention of depression in patients with PD. Especially, there is a systematic review of a study conducted by Wu et al. that reviewed the effectiveness of physical activity on depression in PD patients and also proposed an exercise prescription [21]. Unfortunately, in this study, specific optimization settings for physical activity intervention prescriptions were proposed only through qualitative analysis. Meanwhile, recently, various approaches to augmented reality, virtual reality, and exercises through video games or exergames have been proposed, considering their potential for stimulation, involvement, and engagement also from a cognitive rehabilitation point of view [22,23]. Moreover, new forms of exercise interventions have emerged in recent studies of interventions for depression in patients with PD and have also been shown to be effective, such as resistance exercise [24] and resistance exercise combined with aerobic exercise [25]. To the best of our knowledge, there is no systematic review and meta-analysis in PA intervention for depression in patients with PD. Therefore, this study quantitatively analyzed the effects of physical activity on depression in PD patients by comprehensively searching articles. A subgroup analysis was performed to propose exercise prescriptions to provide an evidence-based basis for future clinical practice of medical professionals in designing exercise programs and improving depression in patients with PD.

## 2. Methods

This systematic review and meta-analysis was performed according to the Preferred Reporting Items for Systematic Reviews and Meta-Analyses (PRISMA 2020) guidelines [26].

### 2.1. Literature Search

Two reviewers independently searched four databases, PubMed, Embase, Cochrane library, and PsycInfo, from the inception of the databases to 22 February 2022. The language was English only, and the search terms were “physical activity”, “Parkinson disease”, and “depression”. The detailed search strategy is described in Supplementary Table S1. In addition, a “carpet” manual search of the references was conducted based on relevant literature. Any disagreements that arose were resolved collaboratively through a third reviewer to reach a final consensus.

### 2.2. Study Selection

Inclusion criteria: (1) patient: subjects diagnosed with Parkinson’s disease; (2) intervention: physical activity; (3) control: nonphysical activity intervention (no intervention, waiting group, etc.); (4) outcome: depression scale data included in the outcome index; (5) study: randomized controlled trials (RCTs).

Scales using the following outcome indicators were included: Beck Depression Inventory (BDI); Hamilton Depression Rating Scale (HAMD); Center for Epidemiological Survey, Depression Scale (CES-D); Profile of Mood States (POMS); Symptom Checklist-90 (SCL-90); Hospital Anxiety and Depression Scale (HADS); Clinical Global Impression (CGI); Quick Inventory of Depressive Symptomology; Self-Report (QIDS-SR); Montgomery–Asberg Depression Rating Scale (MADRS); Self-Rating Depression Scale (SDS); GDS: Geriatric Depression Scale (GDS).

Exclusion criteria: (1) animal experiments, (2) full text not available, (3) meta-analysis/review/conference abstracts/case reports/letters.

Studies combining exercise interventions with other complementary interventions (neurodevelopmental treatments, etc.) were excluded unless the control group also received the same complementary interventions, which would eliminate the effect of the complementary intervention on the effect of the exercise intervention.

### 2.3. Data Extraction

All articles were screened independently by two reviewers for the title, abstract, and full text based on defined predefined criteria, and disagreements were resolved by a third reviewer. Among the data extracted were: first author, year, country/region, age, disease severity, disease duration, sample size, gender and its proportion, intervention type, intervention period, intervention frequency, intervention time, type of control group, outcome indicators, and follow-up time. In addition, for studies where data could not be extracted, we obtained data by contacting the authors.

### 2.4. Quality Assessment

Two reviewers independently assessed the methodological quality of RCT articles using the Cochrane Risk of Bias Assessment Scale [27]. The scale includes seven domains: generation of randomization sequences, allocation protocol concealment, blinding of participants and investigators, blinding of outcome assessors, incomplete outcome data, selective reporting, and other sources of bias. The corresponding judgments of “low risk”, “unknown risk”, and “high risk” were made for each study. Any disagreements were discussed with a third reviewer until the issue was resolved.

### 2.5. Statistical Analysis

Meta-analysis was performed using the Review Manager 5.3 software provided by the Cochrane Collaboration, and statistical significance was defined as a *p*-value < 0.05 (all reported *p*-values are two-sided). The outcome in this study was a continuous variable. Standardized mean differences (SMDs) were calculated as effect sizes for the statistics, and 95% confidence intervals (CIs) were calculated by effect analysis. According to the interpretation of Cohen (1988), SMD < 0.2 is a small effect size, 0.2 ≤ SMD < 0.5 is a small effect size, 0.5 ≤ SMD < 0.8 is a medium effect size, and SMD ≥ 0.8 is a large effect size [28]. The heterogeneity of the study was expressed by I^2^, and the low-, medium-, and high-degree heterogeneities were denoted by the I^2^ statistics of 25%, 50%, and 75%, respectively [29]. A fixed-effects model was used for I^2^ < 50% and *p* ≥ 0.1. A random-effects model was used for I^2^ ≥ 50% and *p* < 0.1. A subgroup analysis by disease duration, intervention type, intervention period, intervention frequency, and intervention time was performed. Meta-regression was performed using the Stata SE 16.0 software (StataCorp, College Station, TX, USA) to explore potential sources of heterogeneity between studies. Funnel plots were used to test for the presence of publication bias. Sensitivity analysis was performed to determine the stability and reliability of the results.

## 3. Results

### 3.1. Characteristics of Included Studies

**Figure 1:**
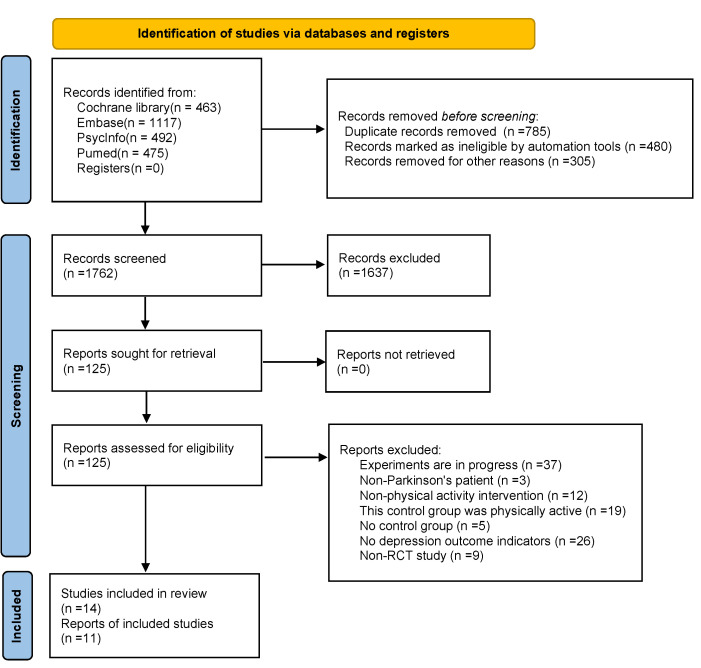
Flow chart with different phases of the search process.

The flow chart of the literature search for the selection process is shown in Figure 1. A total of 2547 articles were found by computer search and manual search, and after screening out duplicate records, 1762 articles remained. Then manual search for titles and abstracts was conducted, 1637 articles were screened out, the remaining 125 articles were screened in full text, and 111 articles were excluded after full-text screening, of which 37 studies were not yet completed; 3 studies had patients that included healthy older adults; 12 studies did not have physical-activity-only interventions; 24 studies did not have a control group, or the control group was physically active; 26 studies did not have depression outcome indicators; and 9 studies were non-RCT types. The final 14 papers agreed by both researchers were included, of which 3 papers [30,31,32] had depression outcome indicators for which data could not be extracted, and only qualitative analysis was performed. The remaining 11 papers [24,25,33,34,35,36,37,38,39,40,41] were subjected to meta-analysis.

The characteristics of the 14 included studies are shown in Table 1. A total of 516 patients with Parkinson’s disease were included, with Hoehn and Yahr scale scores ranging from 1 to 4 and a mean age between 58 and 72 years, of whom 211 were men and 148 were women, and 157 patients from the 4 studies in which gender was not reported [24,33,34,40]. Four studies were conducted in the United States, 3 studies were conducted in Italy, 2 studies were conducted in Korea, and the remaining 5 studies were conducted in Germany, Taiwan, Hungary, Brazil, and Japan. The intervention period was concentrated on 4–26 weeks, the intervention frequency ranged from 1 to 7 per week, and the intervention time range was 10–90 min, mostly concentrated in 30~60 min. The types of physical activity interventions included resistance exercise, aerobic exercise, stretching, yoga, treadmill training, dance, qigong, stationary biking, and walking. The control group was primarily a waiting group for nonexercise interventions, no physical activity interventions, and daily care. Meta-analyses were performed on study patients with multiple inclusions of the same group of patients, but only a small number of patients were involved.

### 3.2. Quality Assessment

**Figure 2:**
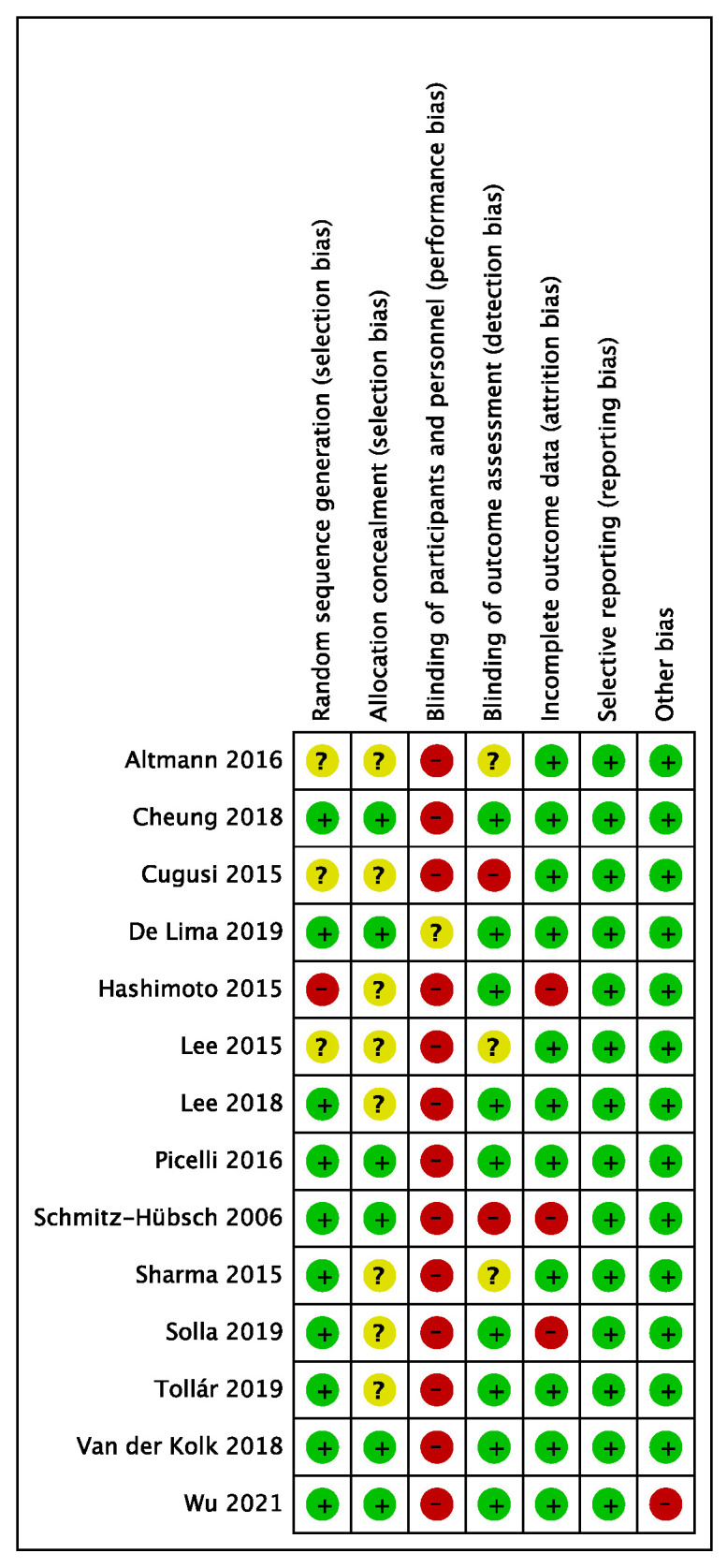
Methodological quality of included studies [24,25,30,31,32,33,34,35,36,37,38,39,40,41].

**Figure 3:**
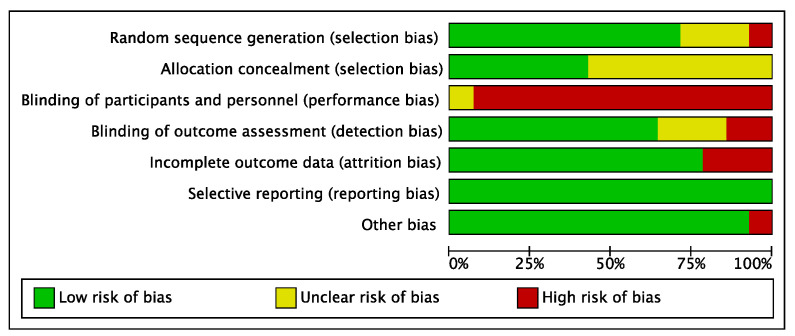
The distribution of the methodological quality of included studies.

The risk of bias assessed for the 14 included studies is shown in Figure 2 and Figure 3. Ten studies [24,25,30,31,32,34,38,39,40,41] described the generation of a completely randomized sequence, and 6 studies [24,25,31,34,38,41] used a suitable allocation concealment method. Thirteen studies [25,30,31,32,33,34,35,36,37,38,39,40,41] described that participants signed an informed consent for the experiment so that participants were nonblinded, which is a common drawback of nondrug clinical trials. There were 9 studies [24,25,32,34,36,38,39,40,41] that were blinded to outcome evaluators. Regarding the completeness of outcome data, participants were missing from 3 studies, with an unbalanced number and reasons for missing between groups [31,36,39]. One study was evaluated for other bias due to the presence of an imbalance between the experimental and control groups at baseline [25].

### 3.3. Effect of the Intervention

**Figure 4:**
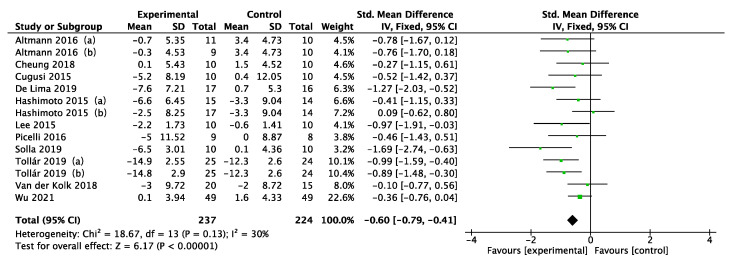
Forest plot of the effect of physical activity on depressive symptoms in PD patients [24,25,33,34,35,36,37,38,39,40,41].

In the 11 studies included in the meta-analysis, a total of five depression scales (BDI, HADS, GDS, HAMD, SDS) were used to measure the effect of physical activity on depression in PD patients. Because the studies took different scales for evaluation, standardized mean difference (SMD) was used as the effect statistic for the analysis. As shown in Figure 4, the fixed effects model revealed a moderate and significant improvement in depression in PD patients who performed physical activity compared with controls (SMD = −0.60; 95% CI = −0.79 to −0.41; *p* < 0.00001). The heterogeneity statistic I^2^ = 30% indicated potential moderate heterogeneity.

### 3.4. Subgroup Analysis

**Figure 5:**
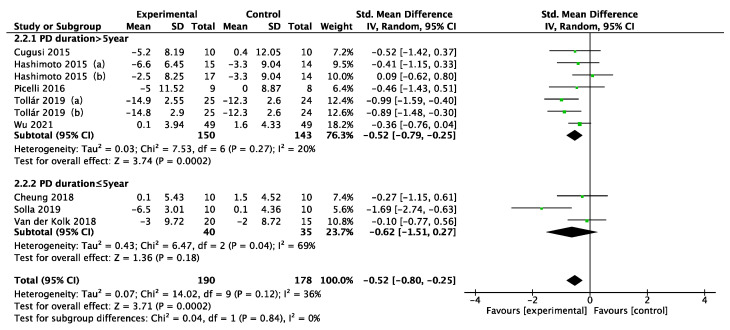
Subgroup analysis of disease duration [25,34,35,36,38,39,40,41].

**Figure 6:**
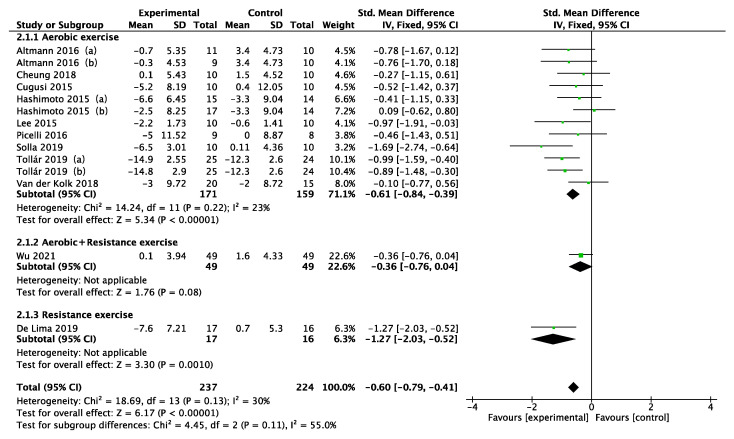
Subgroup analysis of intervention type [24,25,33,34,35,36,37,38,39,40,41].

**Figure 7:**
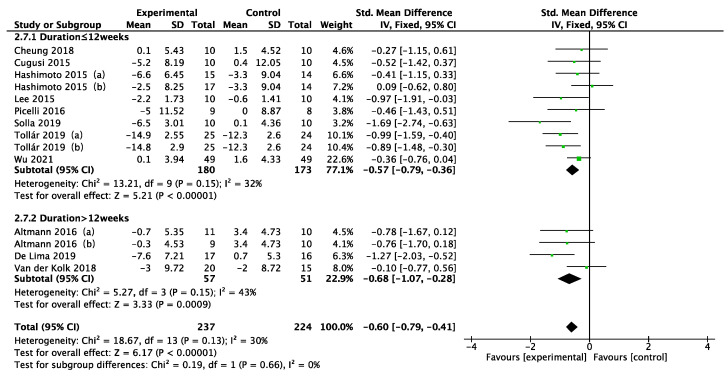
Subgroup analysis of intervention period [24,25,33,34,35,36,37,38,39,40,41].

**Figure 8:**
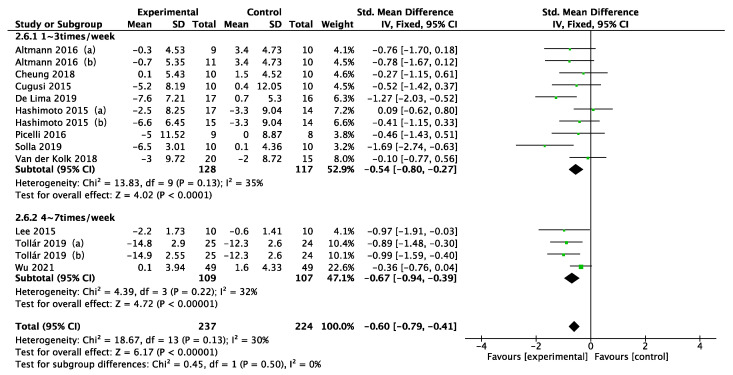
Subgroup analysis of intervention frequency [24,25,33,34,35,36,37,38,39,40,41].

**Figure 9:**
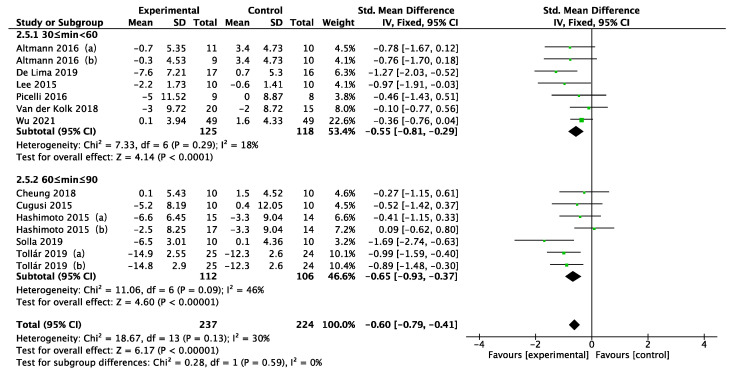
Subgroup analysis of intervention time [24,25,33,34,35,36,37,38,39,40,41].

Disease duration, intervention type, intervention period, intervention frequency, and intervention time were selected for the subgroup analysis in the 11 included studies, as shown in Figure 5, Figure 6, Figure 7, Figure 8 and Figure 9. The results of the subgroup analysis were as follows: (1) disease duration (Figure 5): A subgroup analysis of the duration of Parkinson’s disease revealed that physical activity interventions improved depression in PD patients with disease duration greater than 5 years (SMD = −0.52; 95% CI = −0.79 to −0.25; *p* = 0.0002). (2) Intervention type (Figure 6): For PD patients with depression, the most significant intervention effect was resistance exercise (SMD = −1.27; 95% CI = −2.03 to −0.52; *p* = 0.0010). (3) Intervention period (Figure 7): Performing a physical activity intervention for more than 12 weeks (SMD = −0.68; 95% CI = −1.07 to −0.28; *p* = 0.0009) was superior to conducting an intervention for less than 12 weeks. (4) Intervention frequency (Figure 8): Performing physical activity exercise more than 4 times per week was the best (SMD = −0.67; 95% CI = −0.94 to −0.39; *p* < 0.00001). (5) Intervention time (Figure 9): 60–90 min of physical activity per session significantly improved depression in PD patients (SMD = −0.65; 95% CI = −0.93 to −0.37; *p* < 0.00001).

### 3.5. Regression Analysis

To investigate the sources of heterogeneity in order to identify potential influencing factors. In this study, we searched for influential factors in terms of both exercise intervention programs and basic patient conditions. Six covariates were selected: intervention type, intervention time, intervention frequency, intervention period, age, and disease duration. The results of the regression of the covariates are shown in Table 2. Confidence intervals and *p*-values for each covariate showed no significant effects for intervention type (95% Cl −4.655116 to 3.659473, *p* = 0.729), intervention time (95% Cl −0.4443355 to 0.3350238, *p* = 0.686), intervention period (95% Cl −1.472603 to 1.166919, *p* = 0.737), intervention frequency (95% Cl −4.175988 to 3.244342, *p* = 0.716), age (95% Cl −2.593587 to 2.7119, *p* = 0.948), and disease duration (95% Cl −5.869013 to 5.196527, *p* = 0.859).

### 3.6. Sensitivity Analysis

Sensitivity analysis was performed on the 11 included papers, mainly by excluding the literature and changing the analytical model on a piece-by-piece basis. The test results revealed that the changes were not significant, indicating that the results of the meta-analysis of this study were more stable and reliable. The complete sensitivity analysis is described in Supplementary Table S2.

### 3.7. Publication Bias

**Figure 10:**
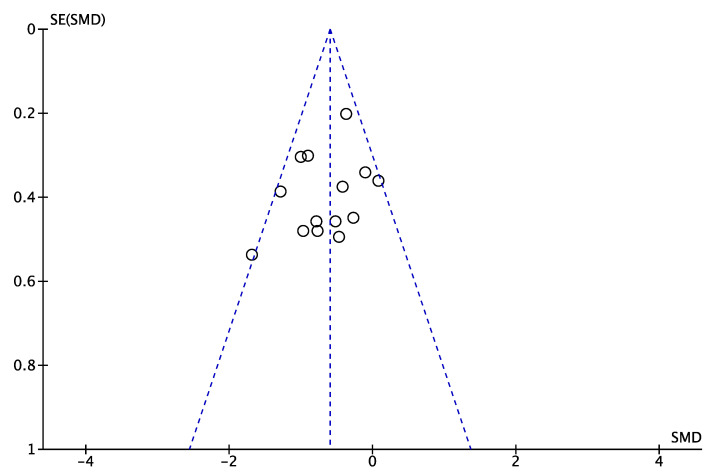
Funnel plots for all included studies.

Because the number of included studies is greater than 10, the funnel plot is used to test, and the test results are shown in Figure 10. From the figure, it can be seen that the scatter distribution is on the upper side, basically balanced between left and right, although there is publication bias in one article on the left side, but it is not very serious and does not have much impact on the results, indicating that the bias results are acceptable and there is no obvious publication bias among the studies.

### 3.8. Adverse Events

None of the 14 trials of the effects of physical activity on PD patients with depression reported adverse events in either the intervention or control groups.

## 4. Discussion

The purpose of this systematic review and meta-analysis was to collate and analyze the effects of RCTs on physical activity on depression in patients with PD, and the results showed that physical activity reduces depression in patients with PD. Subgroup analysis indicated that resistance exercise for 60–90 min more than 4 times per week for up to 12 weeks had a significant effect on PD patients who have had the disease for more than 5 years. Meta-regression showed that intervention type, intervention time, intervention frequency, intervention period, age, and disease duration were not sources of heterogeneity.

The quality assessment of this article was based on the Cochrane Bias Risk Assessment Scale. All of our included RCT experiments showed relatively high methodological quality. This could indicate that the results of our experiments are plausible. We analyzed 11 RCTs on physical activity interventions containing resistance exercise, aerobic exercise, stretching, yoga, treadmill training, dance, stationary cycling, and walking for the treatment of depression in patients with PD. Our experimental results are consistent with those of a number of systematic review-type articles that are similar to the types of physical activity interventions. Ban’s (2021) systematic review shows that yoga is effective in improving depression in PD patients [18]. The Wang (2022) study confirmed the positive effect of dance on depression in PD patients as well [20]. The results of the Song (2017) study showed that exercise modalities regarding tai chi and qigong also showed significant improvements in depression in PD patients [19]. An article in Jin’s (2019) systematic review found that physical and mental exercise slows the development of depression in PD patients [42].

Depression treatment currently faces many difficulties, in large part because of the lack of understanding of clear mechanisms of illness and the large heterogeneity between disorders [43]. The same is true for depression suffered by patients with PD, and the exact mechanism by which depression occurs in PD is not known. However, some studies have suggested that this may be due to (1) alterations in brain structure, such as hippocampal atrophy and hippocampal neuron damage; (2) alterations in neurotransmitter signaling, such as norepinephrine, dopamine, and 5-HT systems, which are known to be affected in PD patients (and these neurotransmitters are also involved in mood regulation); (3) alterations in inflammation and brain-derived neurotrophic factor (BDNF) levels; and (4) psychosocial factors and pain, which may also contribute to the development of depression in PD patients [44]. A large part of the above-mentioned mechanisms that may contribute to the emergence of depression in PD patients is influenced by physical activity, because some studies have found evidence that physical activity may alleviate depression in PD patients by (1) protecting the hippocampal volume of the brain [45] and regenerating hippocampal neurons by decreasing glucocorticoid receptors [46,47]; (2) increasing tryptophan hydroxylase, supplying 5-hydroxytryptamine synthesis [48], promoting dopamine release to improve patients’ mood [49], activating the endogenous cannabinoid system, altering the hypothalamic pituitary–adrenal axis function, and increasing norepinephrine levels [48]; (3) increasing cerebral blood flow, alleviating oxidative stress and inflammation, and increasing the expression of brain-derived neurotrophic factor (BDNF) [50,51]; and (4) reducing disability rates and increasing patients’ quality of life [24]. It is evident that a variety of exercise programs have proven to be valid studies. Therefore, we performed an appropriate subgroup analysis of the physical activity interventions to obtain specific exercise prescriptions.

The results of the subgroup analysis showed that resistance exercise performed more than 4 times per week for 60–90 min for up to 12 weeks was most effective in improving depression in Parkinson’s disease patients with a disease duration of more than 5 years. There are fewer studies on the relationship between depression and disease duration in patients with PD. One longitudinal study showed that depression in PD patients is variable and sustained, with more severe symptoms occurring with increasing disease duration. This leads to a much lower likelihood of symptom remission [52]. Our study found that physical activity was effective for depression in PD patients with a disease duration of >5 years, but not for depression in PD patients with a disease duration of ≤5 years. The reason for the above may be that no investigation of antidepressants was conducted. Depression in early PD patients is easily overlooked, and as the course of the disease increases, PD patients with depression have more severe symptoms and are more likely to take antidepressants. Additionally, the number of interventions in patients with a disease duration of ≤5 years was only 40, and the insufficient sample size may also lead to some bias in the results. A systematic review found that at least 12 weeks of regular physical activity was required to relieve depression in PD patients, which is consistent with our findings that more than 12 weeks of intervention period has a higher effect [53]. The ACSM guidelines for exercise in PD patients recommend that strength and resistance training for at least 30 min 2 to 3 times per week can be effective in improving motor and nonmotor symptoms in PD patients [54]. The results obtained in this study were somewhat different compared with the ACSM guidelines. The difference may be due to the fact that this study only focused on the depression outcome indicators alone and did not focus on other nonexercise symptoms or exercise symptoms of PD. It is worth mentioning that in the subgroup analysis of intervention types, we found the same intervention effect for aerobic exercise + resistance exercise as for the control group. This may be due to the fact that there are fewer studies related to aerobic exercise + resistance group exercise, resulting in biased results and causing no statistical significance. One study showed that as the disease becomes more severe, people with PD often experience a decline in muscle strength and function [55]. The limited mobility leads to a sedentary and socially disconnected state for many patients [50,56]. This sedentary lifestyle and prolonged social isolation in turn lead to an increase in depressive symptoms. The additional positive effect of conducting resistance training compared with other exercise modalities is the improvement of increased muscle strength, increased muscle cross-sectional area, and improved functional capacity in PD patients [57], which in turn improves the efficiency and functional autonomy of daily living activities in PD patients. Ultimately, psychosocial aspects lead to effective improvement of depressive symptoms in PD patients. Nevertheless, in this review, the amount of original literature included on different exercise types cannot be ignored. Even so, resistance exercise had the largest effect sizes in the subgroup analysis of intervention types and there is research demonstrating the additional benefits of resistance exercise compared with other exercise modalities in depressed patients. However, only 1 study of resistance exercise was included in this study, which may lead to a slight limitation of the effect of resistance training due to the lack of available studies. It is of interest to note that aerobic exercise interventions, based on the findings of 9 existing studies, also achieved large and significant effect sizes and provided as much effectiveness as resistance training. Our study concludes that the results of aerobic exercise are more reliable, while the effects of resistance training will require more research to prove its effectiveness in the future. As it stands, the type of aerobic exercise intervention may be used by medical professionals as a primary or adjunctive intervention for clinical rehabilitation in PD patients with depression.

Regarding heterogeneity in this study, the meta-regression analysis taken found that age, disease duration, intervention type, intervention time, intervention frequency, and intervention period were not sources of heterogeneity. It is noteworthy that of the 11 studies included, none used specific inclusion criteria to ensure that the PD patients participating in the experiment had depression. By looking at the depression scores (mean and standard deviation) at baseline for the included experiments, it was found that participants who completed the depression scale before the exercise intervention had scored either above or below the cut-off point for a diagnosis of depression, suggesting that there were participants with and without depression among the recruited patients. This situation may increase the heterogeneity of the results. Another potential source of heterogeneity may be the use of different depression scales to measure depression. Seven of the studies used the Beck Depression Inventory (BDI) [33,34,35,37,38,39,40]; the remaining 4 studies used the Geriatric Depression Scale (GDS) [25], Hospital Anxiety and Depression Scale (HADS) [41], Hamilton Depression Rating Scale (HAMD) [24], and Self-Rating Depression Scale (SDS) [36] to measure depression scores. The article of Solla (2019) [39] may also be one of the sources of heterogeneity in this review. By looking into the forest plot (Figure 4), it was found that this article had the greatest impact on PD patients with depression compared with other interventions, while in sensitivity analysis, heterogeneity was found to be reduced from 30% to 17% by excluding the literature of Solla (2019). The research analysis revealed that the intervention model adopted in this study is a Sardinian folk dance commonly known as ballu sardu (BS). This traditional dance differs from conventional physical activity in that it is a multisensory experience, not just a set of isolated movements driven by music, but also involves cognitive, emotional, cultural, and socioethnic dance aspects [58]. Because the features of BS dance involve physical activity and cognitive functioning and social aspects, BS dance may be a good treatment for PD patients suffering from depression. However, there are currently no studies comparing ballu sardu dance with other dance interventions (e.g., virtual reality dance) or physical activity intervention programs in PD patients. In future studies, high-quality experiments can be conducted to compare the effects of interventions on depression in PD patients.

There are several limitations to this study, which are as follows: First, the literature search only included articles in the English language, and some experiments reported in other languages may have been missed. Second, there was only one de Lima (2019) study [24] on resistance exercise in the included literature, and conclusions drawn from a small number of studies may be somewhat biased, and more high-quality literature on resistance exercise should be included in future studies to corroborate this finding. Third, because the included literature did not report on the use of antidepressants, we did not assess the difference between the use of medication and the nonuse of medication. Finally, the experimental sizes of the included studies in the meta-analysis were relatively small, with only 2 of the 11 studies having more than 50 participants, while the duration of postexercise improvements could not be assessed due to limited data. Incidentally, the lack of registration in this study may lead to limitations in methodological transparency.

## 5. Conclusions

The results of this meta-analysis show that physical activity has a positive effect on depression in patients with PD. Although this paper found the optimal exercise prescription through 11 RCTs, due to the limited amount of literature and experimental design, other larger sample sizes and high-quality studies are needed to validate these effects in the future.

## Figures and Tables

**Table 1:** Characteristics of the studies included in the study.

Main Author	Year	Country/ Region	Age	H and Y	PD Duration	Sample Size (M/F)	Experimental Group Intervention (Duration/Frequency/Time)	Control Group	Depression Outcome Measures	Follow-Up Time (Week)
de Lima [24]	2019	Brazil	66.7 ± 5.3	1–3	No reported	33 (no reported)	**Type:** resistance training (20 weeks × 2/week × 30–40 min/session)	No exercise	HAMD	20 weeks
Altmann [33]	2016	USA	64.6 ± 8.7	1–3	No reported	30 (no reported)	**Type:** G1: aerobic exercise; G2: stretch-balance training (16 weeks × 3/week × from 20 min/session to 45 min by increasing exercise time by 5 min each week)	Continued normal activities	BDI	16 weeks
Cheung [34]	2018	USA	63 ± 8.0	1–3	4.8 ± 2.9	20 (no reported)	**Type:** yoga (12 weeks × 2/week × 60 min/session)	Wait list	BDI	26 weeks
Cugusi [35]	2015	Italy	67.3 ± 7.8	1–3	7.0 ± 3.0	20 (16/4)	**Type:** Nordic walking (12 weeks × 2/week × 60 min/session)	Conventional care	BDI	12 weeks
Hashimoto [36]	2015	Japan	66.5 ± 10.4	2–4	7.0 ± 5.0	46 (12/34)	**Type:** G1: dance; G2: PD exercise (12 weeks × 1/week × 60 min/session)	No intervention	SDS	12 weeks
Lee [37]	2015	Korea	69.3 ± 3.1	No reported	No reported	20 (10/10)	**Type:** virtual reality dance (6 weeks × 5/week × 30 min/session)	No exercise	BDI	6 weeks
Picelli [38]	2016	Italy	71.4 ± 8.1	1–3	11.0 ± 4.8	17 (12/5)	**Type:** treadmill training (4 weeks × 3/week × 45 min/session)	Regular social interactions	BDI	4 weeks
Solla [39]	2019	Italy	67.4 ± 6.1	1–3	4.7 ± 3.7	20 (13/7)	**Type:** Sardinian folk dance (12 weeks × 2/week × 90 min/session)	Usual care	BDI	12 weeks
Tollár [40]	2019	Hungary	69.4 ± 4.5	2–3	7.4 ± 2.0	74 (no reported)	**Type:** G1: agility exergaming; G2: stationary cycling (5 weeks × 5/week × 60 min/session)	Wait list	BDI	5 weeks
van der Kolk [41]	2018	USA	58.9 ±8.8	1–2	0.92 ± 0.9	37 (24/13)	**Type:** aerobic exercise(stationary bike) (26 weeks × 3/week × 30 min/session)	No intervention	HADS	26 weeks
Wu [25]	2021	Taiwan	65.1 ±7.5	1–2	5.3 ± 3.9	98 (56/42)	**Type:** home-based exercise (aerobic exercise, resistance training) (8 weeks × >3/OR7/week × 30–50 OR 10–15 min/session)	No exercise	GDS	8 weeks
Lee [32]	2018	Korea	65 ± 6.8	1–3	No reported	32 (17/15)	**Type:** Turo (qi dance) (8 weeks × 2/week × 60 min/session)	Wait list	BDI	8 weeks
Sharma [30]	2015	USA	66.9 ± 12.0	1–2	3.0 ± 0.4	13 (8/5)	**Type:** yoga (12 weeks × 2/week × 60 min/session)	No intervention	GDS	12 weeks
Schmitz-Hübsch [31]	2006	Germany	63.8 ± 7.5	No reported	5.8 ± 4.2	56 (43/13)	**Type:** qigong (8 weeks × 1/week × 60 min/session)	No intervention	MADRS	52 weeks

H and Y: Hoehn and Yahr scale; PD: Parkinson’s disease; M: male; F: female; BDI: Beck Depression Inventory; HADS: Hospital Anxiety and Depression Scale; HAMD: Hamilton Depression Rating Scale; SDS: Self-Rating Depression Scale; GDS: Geriatric Depression Scale.

**Table 2:** Regression analysis of covariate for PD patients with depression.

_ES	Coef.	Std. Err.	t	*p* > |t|	95% Conf. Interval	
Type	−0.4978215	1.30632	−0.38	0.729	−4.655116	3.659473
Time	−0.0546558	0.1224466	−0.45	0.686	−0.4443355	0.3350238
Period	−0.1528421	0.4147001	−0.37	0.737	−1.472603	1.166919
Frequency	−0.4658228	1.165822	−0.40	0.716	−4.175988	3.244342
Age	0.0591567	0.8335549	0.07	0.948	−2.593587	2.7119
Disease duration	−0.336243	1.738527	−0.19	0.859	−5.869013	5.196527
_Cons	4.253626	29.36428	0.14	0.894	−89.19663	97.70389

## Data Availability

The data used to support the findings of this study are included in the article.

## Supplementary Materials

The following supporting information can be downloaded at: https://www.mdpi.com/article/10.3390/ijerph19116849/s1: Table S1: Search strategy; Table S2: Sensitivity analysis.
