# Supplementary material for: Effect of Physical Activity on Depression in Patients with Parkinson’s Disease: A Systematic Review and Meta-Analysis
Source: Int J Environ Res Public Health. 2022 Jun 3;19(11):6849. doi: 10.3390/ijerph19116849 (PMC9180645; doi:10.3390/ijerph19116849)
Supplement: Supplementary file 1 [file ijerph-19-06849-s001.zip › ijerph-1731835-supplementary/Supplementary Table S1. Search Strategy.pdf]

1.Physical Activity：

physical activity OR exercise OR fitness OR training OR sport* OR energy expenditure OR caloric expenditure OR physical education OR cycling* OR jogging OR Walking OR running OR Qigong OR Tai Chi OR yoga OR exercising OR exercises

2.Parkinson Disease：

Idiopathic Parkinson's Disease OR Lewy Body Parkinson's Disease OR Parkinson's Disease, Idiopathic OR Parkinson's Disease, Lewy Body OR Parkinson Disease, Idiopathic OR Parkinson's Disease OR Idiopathic Parkinson Disease OR Lewy Body Parkinson Disease OR Primary Parkinsonism OR Parkinsonism, Primary OR Paralysis Agitans OR PD

3. Depression：

Depression OR Depressive Symptoms OR Depressive Symptom OR Symptom, Depressive OR Symptoms, Depressive OR Emotional Depression OR Depression, Emotional OR Depressive Disorder OR Depressive Disorders OR Disorder, Depressive OR Disorders, Depressive OR Neurosis, Depressive OR Depressive Neuroses OR Depressive Neurosis OR Neuroses, Depressive OR Depression, Endogenous OR Depressions, Endogenous OR Endogenous Depression OR Endogenous Depressions OR Depressive Syndrome OR Depressive Syndromes OR Syndrome, Depressive OR Syndromes, Depressive OR Depression, Neurotic OR Depressions, Neurotic OR Neurotic Depression OR Neurotic Depressions OR Melancholia OR Melancholias OR Unipolar Depression OR Depression, Unipolar OR Depressions, Unipolar OR Unipolar Depressions
