# Supplementary material for: Effect of Physical Activity on Depression in Patients with Parkinson’s Disease: A Systematic Review and Meta-Analysis
Source: Int J Environ Res Public Health. 2022 Jun 3;19(11):6849. doi: 10.3390/ijerph19116849 (PMC9180645; doi:10.3390/ijerph19116849)
Supplement: Supplementary file 1 [file ijerph-19-06849-s001.zip › ijerph-1731835-supplementary/Supplementary Table S2. Sensitivity analysis.pdf]

**Sensitivity Analysis**	**Heterogeneity test**	**Effect size：SMD（95%CI）**
**Previous result**	Heterogeneity: $Chi^2 = 18.67$, df = 13 (P = 0.13); $I^2 = 30\%$	-0.60 [-0.79, -0.41]
**Method 1：**		
Changing the analysis model	Heterogeneity: $Tau^2 = 0.06$; $Chi^2 = 18.67$, df = 13 (P = 0.13); $I^2 = 30\%$	-0.62 [-0.86, -0.39]
**Method 2：**		
Altmann 2016 excluded	Heterogeneity: $Chi^2 = 18.37$, df = 11 (P = 0.07); $I^2 = 40\%$	-0.58 [-0.78, -0.38]
Cheung 2018 excluded	Heterogeneity: $Chi^2 = 18.11$, df = 12 (P = 0.11); $I^2 = 34\%$	-0.61 [-0.81, -0.42]
Cugusi 2015 excluded	Heterogeneity: $Chi^2 = 18.64$, df = 12 (P = 0.10); $I^2 = 36\%$	-0.60 [-0.79, -0.41]
De Lima 2019 excluded	Heterogeneity: $Chi^2 = 15.40$, df = 12 (P = 0.22); $I^2 = 22\%$	-0.55 [-0.75, -0.36]
Hashimoto 2015 excluded	Heterogeneity: $Chi^2 = 14.33$, df = 11 (P = 0.22); $I^2 = 23\%$	-0.67 [-0.87, -0.46]
Lee 2015 excluded	Heterogeneity: $Chi^2 = 18.04$, df = 12 (P = 0.11); $I^2 = 33\%$	-0.58 [-0.77, -0.39]
Picelli 2016 excluded	Heterogeneity: $Chi^2 = 18.59$, df = 12 (P = 0.10); $I^2 = 35\%$	-0.60 [-0.80, -0.41]
Solla 2019 excluded	Heterogeneity: $Chi^2 = 14.41$, df = 12 (P = 0.28); $I^2 = 17\%$	-0.56 [-0.75, -0.37]
Tollár 2019 excluded	Heterogeneity: $Chi^2 = 15.34$, df = 11 (P = 0.17); $I^2 = 28\%$	-0.51 [-0.72, -0.30]
Van der Kolk 2018 excluded	Heterogeneity: $Chi^2 = 16.42$, df = 12 (P = 0.17); $I^2 = 27\%$	-0.64 [-0.84, -0.44]
Wu 2021 excluded	Heterogeneity: $Chi^2 = 16.92$, df = 12 (P = 0.15); $I^2 = 29\%$	-0.67 [-0.88, -0.45]
